# Numerical Modeling and Evaluation of PEM Used for Fuel Cell Vehicles

**DOI:** 10.3390/ma14247907

**Published:** 2021-12-20

**Authors:** Yousef Darvishi, Seyed Reza Hassan-Beygi, Payam Zarafshan, Khadijeh Hooshyari, Urszula Malaga-Toboła, Marek Gancarz

**Affiliations:** 1 Department of Biosystems Engineering, University of Tehran, Tehran P.O. Box 113654117, Iran; sdarvishi@ut.ac.ir; 2 Department of Agrotechnology, College of Abouraihan, University of Tehran, Tehran P.O. Box 113654117, Iran; p.zarafshan@ut.ac.ir; 3 Department of Applied Chemistry, Faculty of Chemistry, Urmia University, Urmia P.O. Box 5756151818, Iran; kh.hooshyari@urmia.ac.ir; 4 Faculty of Production and Power Engineering, University of Agriculture in Krakow, Balicka 116B, 30-149 Krakow, Poland; umalagatobola@gmail.com; 5 Institute of Agrophysics Polish Academy of Sciences, Doświadczalna 4, 20-290 Lublin, Poland

**Keywords:** numerical modeling, fuel cell system, polymer membrane, FCV

## Abstract

The present study sought to analyze a novel type of polymer membrane fuel cell to be used in vehicles. The performance of the fuel cell was evaluated by modeling the types of production–consumption heat in the anode and cathode (including half-reaction heat, activation heat, and absorption/desorption heat) and waterflood conditions. The meshing of flow channels was carried out by square cells and the governing equations were numerically discretized in the steady mode using the finite difference method followed by solving in MATLAB software. Based on the simulation results, the anodic absorption/desorption heat, anodic half-reaction heat, and cathodic activation heat are positive while the cathodic absorption/desorption heat and cathodic half-reaction heat show negative values. All heat values exhibit a decremental trend over the flow channel. Considering the effect of relative humidity, the relative humidity of the cathode showed no significant change while the anode relative humidity decreased along the flow channel. The velocity at the membrane layer was considerably lower, due to the smaller permeability coefficient of this layer compared to the gas diffusion and reactants (cathode) layers.

## 1. Introduction

Energy and its corresponding approaches are among the most serious challenges in the 21st century. Nowadays, the application of novel sources of energy has been highly considered due to global warming, air pollution, population growth, increasing fossil fuel prices, depletion of fossil fuel resources, and environmental problems. New energy sources should have features such as easy access, renewability, causing no environmental pollution, high energy density, low cost, ease of storage, and economical portability. However, a narrow class of energies currently falls into this category. Fuel cell energy is among the types of energy that has been extensively explored in recent years. A fuel cell is a battery-like device that generates electrical energy. The hydrogen requirement of the fuel cell can be supplied from various sources, such as petroleum products, coal, water electrolysis, biogas, natural gas, and biomass-produced syngas. In a fuel cell, hydrocarbons do not burn directly, and electricity is rather generated by the interactions of hydrogen and oxygen in the air in a combustion-free process. In this regard, electrical energy with high efficiency can be generated by the direct combination of the fuel and oxidizer without noise and environmental pollution. Direct electricity generation is a proper alternative to the Carnot cycle for converting the chemical energy of fossil fuels into mechanical and, ultimately, electrical energies [1,2,3,4,5,6,7,8,9,10,11,12]. 

Modeling the behavior of proton exchange membrane fuel cells (PEMFC) is generally accompanied by complexities. A better understanding of the behavior of PEMFC requires the determination of the membrane proton conductivity in terms of its structural properties. Most modeling approaches examine the entire fuel cell system, including all its components such as anode, cathode, membrane, catalytic layers, gas diffusion layers, and flow channels. So far, a large body of research has been devoted to the simultaneous reduction of the limitations and costs. Despite the significant improvements in overall fuel cell performance in recent years, the main challenge is water transfer along with heat transfer [12,13,14,15,16,17,18]. A two-phase model was proposed for a high-temperature PEMFC fuel cell to improve its modeling. The proposed model considered all polarization and transfer phenomena in good agreement with laboratory data in the temperature range of 150–170 °C. A parametric investigation was carried out to explore the dependence of fuel cell efficiency on membrane doping level, catalyst activity, and transfer characteristics of gases dissolved in the electrolyte medium. Important transfer limitations were found in both electrodes at the catalyst level of 0.1–1%. Notably, the high temperature of the fuel cell may dry the membrane reduces ionic conductivity and increasing thermal stresses, leading to membrane rupture. Low temperatures, however, decelerate the reactions and increase the loss. Further, a decrease in the cell temperature declines the saturation pressure, intensifies water distillation, and causes flotation. Therefore, the fuel cell is faced with temperature restrictions in both extremes of high and low temperatures. Moreover, lower cell temperature variations are desirable [19]. Moreover, other important issues, such as starting the fuel cell in the vehicle under ambient conditions, different operating conditions of the vehicle due to large changes in fuel cell temperature, the low temperature difference between the fuel cell and the environment compared to the internal combustion engines, the difficulty of cooling the fuel cell, and the preference of using low-moisture inlet gases to consume less power for humidifying the inlet gases [20,21], necessitate the study of the role of input conditions in the fuel cell performance functional variables, such as flow rate, in the vehicle.

A large number of studies have been performed on different types of fuel cells to assess the fuel cell performance, including the characteristics of the cell membrane and electrochemical poles, as well as the effect of inlet temperature, pressure, and humidity [22,23,24,25]. The analysis of the fuel cell system of the vehicle includes examining the use of different fuels instead of hydrogen, investigating hybrid systems based on the fuel cells, studying the effect of functional variables such as temperature and pressure on the performance of systems, and optimizing systems to reduce price and weight. Other studies have addressed the current-voltage diagrams of the fuel cell for its application in the vehicle [26,27]. These studies examined the entire fuel cell, and each cell piece was not explored in detail, due to the system complexities, heavy calculations, and the effect of hard masses on each other. Consequently, researchers have been forced to impose restricting assumptions, such as single-phase or constant cell temperature. Lobato et al. examined the effect of flow channel geometry of a PEMFC using a three-dimensional model considering three different geometries of four-stage helical, parallel, and pin-type for the flow channel. Each geometry led to a specific current density profile, suggesting the direct dependence of the flow density distribution on the diffusion of the reactant at the electrode surface [27]. The model predicts the lower efficiency of parallel flow channels due to the presence of a preferential path, preventing the proper distribution of reactive gases over the entire electrode surface. This study also addressed the effect of the intensity of gas inlet flows and temperature. The model proposed in the study predicted better fuel cell performance at higher temperatures [28,29,30,31,32,33,34,35,36]. A one-dimensional dynamic isotherm model was proposed for the transient and steady behavior of polymer electrolyte membrane fuel cells (PEMFC). This model considered the transient mass transfer of components in bipolar plates and gas diffusion layers, as well as the filling and emptying of electric double layers [37,38,39,40,41,42,43,44,45,46,47,48].

In this study, the steady-state behavior of the fuel cell with membranes based on sulfone polymers and the resistance spectra were obtained. Therefore, accurate mathematical modeling is required to predict cell performance and water and heat management in a polymer fuel cell. Furthermore, the effect of various parameters (such as relative humidity, pressure, temperature, etc.) on the performance of a fuel cell could be studied by accurate mathematical modeling. The aim of present study was to introduce suitable model and optimal membrane proton conductivity conditions as a function of different variables and heat and water management in a polymer fuel cell with membranes based on sulfone polymers [49,50,51,52,53,54,55,56,57,58,59,60,61,62,63,64].

## 2. Materials and Methods

### 2.1. Polymer Membrane Fuel Cell

**Figure 1:**
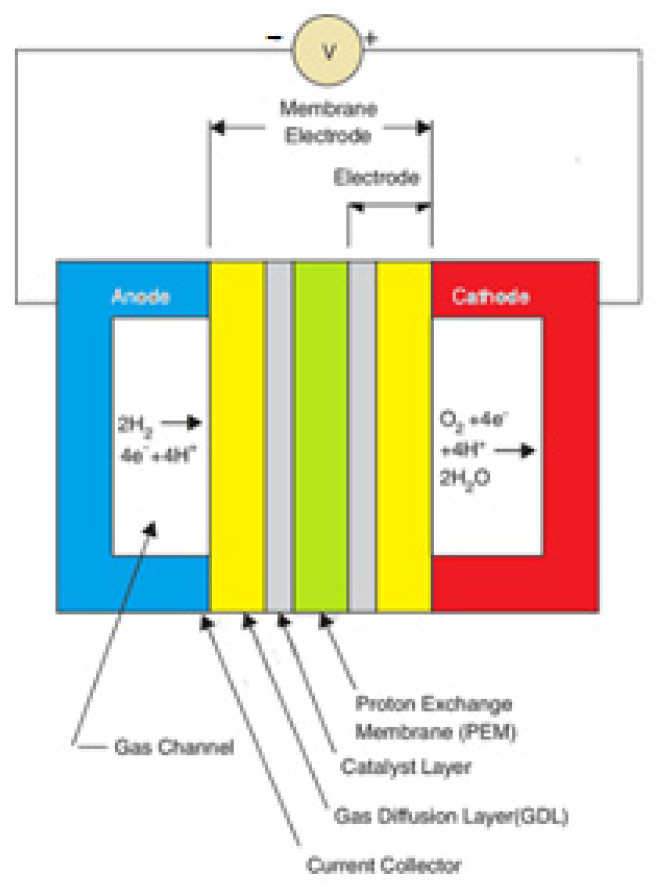
Overview of a PEM fuel cell.

Numerous studies have addressed the industrial and commercial production of polymer electrolyte membrane (PEM) fuel cells. Most of these studies uses renewable energy sources as fuel to minimize pollution. The membrane of this type of cell is made of polymer and its function is to transfer the protons produced in the anode to the cathode. Therefore, this membrane should have high proton conductivity. The PEM fuel cell operates as follows: Fuel, which can be hydrogen, methanol solution, ethanol, and formic acid, enters the anode and oxidizes into protons and electrons (in the case of hydrogen, otherwise to some other products such as carbon dioxide). Figure 1 illustrates a PEM fuel cell.

The hydrogen oxidation reactions that take place at the anode of the PEM fuel cell are presented in Equation (1):

(1) $$H_{2}\Rightarrow 2H^{+}+2e$$

After the oxidation reaction, the proton travels to the cathode through the polymer membrane while the electron travels to the cathode through the external circuit. By blowing oxygen or air at the cathode side, the following reduction reaction occurs on the surface of the cathode catalyst, leading to water production.

(2) $$\frac{1}{2}O_{2}+2H^{+}+2e\Rightarrow H_{2}O$$

The lifespan of this type of cell is more than $5\times 10^{5}$ h (an increase in the temperature of the cell decreases its lifespan) and the output current density is higher than that of all known cells. Hence, the PEM fuel cell is mostly used for vehicles and small electrical systems. In general, about 50% of its maximum power is quickly available at room temperature. This cell can reach its full power under normal conditions after 3 min. The heat from the fuel cell can be exploited for air-conditioning or water heating [19].

### 2.2. Fuel Cell Vehicle System

**Figure 2:**
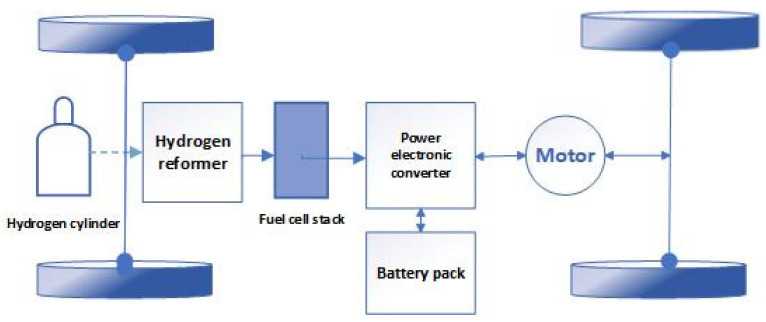
Schematic of a fuel cell vehicle system.

A fuel cell alone could not generate sufficient mechanical power in a vehicle. Thus, auxiliary equipment is required. Figure 2. demonstrates the outputs of the fuel cell system. Receiving hydrogen gas and air as input, a fuel cell produces heat and electrical energy through a chemical process. The electrical energy generated in this process is of the direct current (DC) type and is not suitable for launching engines. Therefore, the DC power should be converted to the alternating current (AC) through an inverter. Then, the vehicle can be steered by engines that are directly connected to the wheels. These vehicles use electrical engines which operate on AC power.

### 2.3. Mathematical Equations

#### 2.3.1. Equations Governing the Transport of Various Species

**Figure 3:**
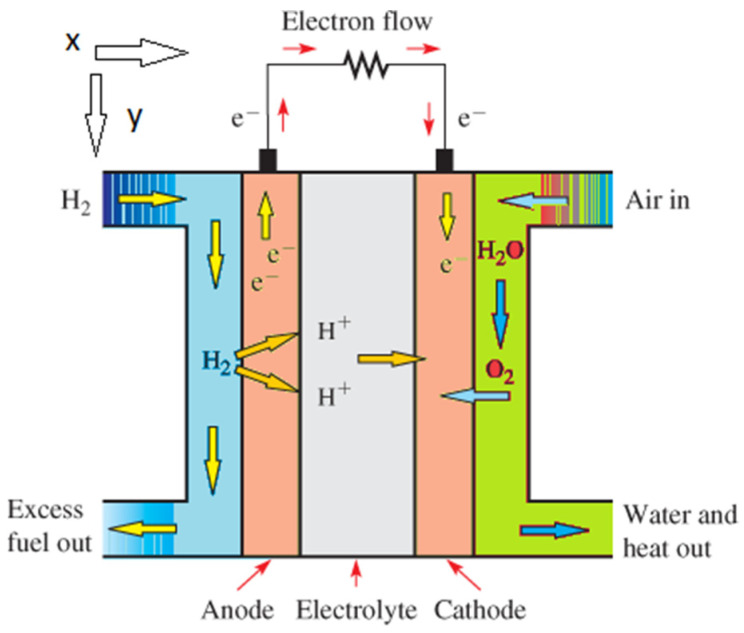
Schematic of a cell in a fuel cell.

In a polymeric fuel cell, hydrogen and oxygen/air are fed to the anode and cathode flow channels, respectively. The molar rates of hydrogen and oxygen input to the anode and cathode flow channels can be obtained by Equations (3) and (4):

(3) $$\dot{{\dot{N}}_{O_{2}}^{cell}}=\frac{S_{C}A_{cell}\bar{I}}{4F}$$

(4) $$\dot{{\dot{N}}_{H_{2}}^{cell}}=\frac{S_{a}A_{cell}\bar{I}}{2F}$$

where $S_{a}$ and $S_{c}$ represent the stoichiometric coefficients of anode and cathode, respectively. $A_{cell}$ denotes the cross-section, while I demonstrates the current density and F is the Faraday constant. Further, $\dot{{\dot{N}}_{H_{2}}^{cell}}\;\mathrm{and}\;\dot{{\dot{N}}_{O_{2}}^{cell}}$ are molar flow rates of hydrogen and oxygen entering the flow channels, respectively. The current density in a polymer fuel cell under a steady state is determined by the transport rate of the species participating in the reaction. In other words, the current density can be determined based on the transport rate of oxygen and hydrogen from the cathode and anode flow channels to reaction sites [20]. Given that the cross-section dimensions of the anode and cathode flow channels are in millimeter scale, the concentration gradients created for different types of the gas phase in the y,x directions are small and negligible. Therefore, the concentration gradients for different types of the gas phase were only considered in the direction x. Consider a point like x in Figure 3, at which the relationship between the production/consumption rates of the species participating in the reaction and the local current density, I, can be obtained using the following equation.

(5) $$\frac{dN_{i}}{Dx}=\xi_{i}\frac{wI\left(x\right)}{4F}$$

where $N_{i}$ represents the molar flow rate of species *i*, w denotes the channel width, I(x) shows the local current density, and $\xi_{i}$ is the stoichiometric parameter of the local species [20,21,22,23,24,25,26].

#### 2.3.2. Flood State Equations

The amount of liquid water generated in a polymer fuel cell and the formation of flooding state depend on the difference between the partial pressure of water vapor $P_{W}^{V}$ and the water saturation pressure $P_{W}^{sat}$ within the cell. Water vapor condensation occurs in the cavities of the gas flow distributor if the partial pressure of water vapor exceeds the saturation pressure of water. Similarly, if the partial pressure of water vapor is lower than the water saturation pressure, the liquid water inside the cell evaporates. Therefore, the emergence of flooding state and two-phase conditions in the anode/cathode flow channels of a polymer fuel cell depends on the amount of liquid water inside the flow channels and water vapor flux entering and leaving the membrane, which can be determined by the following equations [32,33].

(6) $$\frac{{dN}_{w,k}^{i}}{dx}=\left(\frac{k_{c}wh}{RT}\right)\left(P_{W}^{V}-P_{w.k}^{sat}\right)$$

(7) $$\frac{{dN}_{w,a}^{v}}{dx}=-\frac{{dN}_{w,a}^{L}}{dx}-w\frac{aI\left(x\right)}{F}$$

(8) $$\frac{{dN}_{w,c}^{v}}{dx}=-\frac{{dN}_{w,c}^{l}}{dx}+w\frac{\left(1+s\alpha \right)I\left(x\right)}{2F}$$

where v and l represent the vapor and liquid phase index, respectively. w shows the water index, *R* demonstrates the universal gas constant, h denotes the channel height, and *k* is the homogeneous rate constant for water condensation/evaporation [24]. The water vapor condensation process occurs at the interface between the catalytic layer and the gas diffusion layer (GDL) and leads to the formation of liquid droplets in the porous space of the layer. By reaching the common boundary of the GDL-flow channel, these liquid droplets move along the airflow. Regarding capillary forces, liquid water moves inside the porous space of the gas diffusion layer. Using Darcy’s law, it is possible to obtain the velocity of liquid water inside the gas flow distributor layer $N_{w}^{i}$:

(9) $$v_{i}=-\frac{k_{w}}{\mu w}\nabla P_{i}$$

(10) $$N_{w}^{i}=-\frac{p_{w}k_{w}}{M_{w}\mu_{w}}\nabla P_{I}$$

where $P_{I}$ denotes the liquid water pressure, $k_{w}$ indicates the permeability of liquid water in the GDL, μw shows liquid water viscosity, $p_{w}$ stands for the liquid water density, and $M_{w}$ is the water molecular weight [39].

#### 2.3.3. Equations Governing Cell Electrochemistry

The output voltage of a polymer fuel cell, $E_{cell}$, can be obtained based on the difference between the amount of potential drop in the cell and the open-circuit voltage of the cell as follows:

(11) $$E_{cell}=E_{oc}-\eta_{act}-\eta_{ohm}-\eta_{conc}$$

where $E_{oc}$ represents the open-circuit voltage (v). Moreover, $\eta_{act},\;\eta_{ohm},$ and $\eta_{conc}$ are the activation, ohmic, and concentration overpotentials, respectively. The amount of open-circuit voltage can be obtained by Equation (12):

(12) $$E_{oc}=1.229-0.85\times 10^{-3}\left(T-298.15\right)+4.31\times 10^{-5}T\left({\ln P}_{H_{2}}+0.5{\ln P}_{O_{2}}\right)$$

where $P_{H_{2}}$ and $P_{O_{2}}$ are partial pressures of hydrogen and oxygen (Pa), respectively. By neglecting the amount of activation overpotential of the anode, it is possible to use Equation (13) to reduce the amount of activation overpotential of the cathode:

(13) $$\eta_{act}\left(x\right)=\frac{RT}{0.5F}\ln\left(\frac{I\left(x\right)}{I_{0}P_{O_{2}}^{cat}\left(x\right)}\right)$$

where $I_{0}$ represents the exchange current density in a reference pressure (A/m^2^) and $P_{O_{2}}^{cat}$ denotes the partial pressure of oxygen in the catalytic layer (Pa) [37]:

(14) $$P_{O_{2}}^{cat}\left(x\right)=C_{O_{2}}^{cat}\left(x\right)RT$$

where $C_{O_{2}}^{cat}$ stands for the oxygen concentration at the catalyst level, which is related to the oxygen concentration in the flow channel $C_{O_{2}}^{bulk}\left(x\right)$ as follows:

(15) $$C_{O_{2}}^{cat}\left(x\right)=C_{O_{2}}^{bulk}\left(x\right)-\frac{I\left(x\right)}{4F}\left(\frac{1}{h_{O_{2}}}+\frac{t_{GDL}}{D_{O_{2}-g}^{eff}}\right)$$

In the above equation, $h_{O_{2}}$ represents the oxygen mass transfer coefficient whereas $D_{O_{2}-g}^{eff}$ shows the oxygen-effective diffusion coefficient in the gaseous mixture. The latter should be used to transfer oxygen within the porous space of the gas diffusion layer. The cross-sectional area of anode and cathode flow channels of polymer fuel cells have a square shape and the fluid flow regime within these channels is of steady type.

The membrane conductivity can be obtained using Equation (16):

(16) $$k_{m}\left(x\right)=\left(0.5139\lambda_{m}-0.3260\right)\exp\left[1268\left(\frac{1}{303}-\frac{1}{T}\right)\right]$$

The mean current density in a polymer fuel cell at a given voltage, $\bar{I}$, can be obtained by integrating the local current density, I(x), along the flow channel.

(17) $$\bar{I}=\frac{1}{L}\int_{O}^{L}\left[1-\bar{S}\left(x\right)\right]I\left(x\right)dx$$

#### 2.3.4. Equations Governing Thermal Energy

Regarding the millimetric dimension of the cross-section of the anode and cathode flow channels, the temperature gradients in y and z directions of the channel are negligible. The general equation governing thermal energy in a flow channel of a polymeric fuel cell is as follows [37,38,39,40,41]:

(18) $$p_{mix}C_{p,mix}v_{x}\frac{\partial T}{\partial x}=k_{mix}\frac{\partial^{2}}{\partial x^{2}}+Q_{total}^{source}$$

where $p_{mix}$ and $C_{p,mix}$ represent the density and heat capacity of the gaseous mixture, respectively. $v_{x}$ depicts the gas flow velocity in *x* direction, and $k_{mix}$ shows the gas mixture thermal conductivity. Furthermore, T is the gas flow temperature and Qsource deotes the entire heat energy (exchanged, generated, or consumed). Regarding the reactions occurring in a polymeric fuel cell, it is assumed that the produced water is liquid. Thus, the absorption/desorption heat of water molecules should be taken into account at the electrode surface. The balance between the liquid (absorbed) and the gas (excreted) phases at their interface controls the absorption phenomenon. The enthalpy change related to the absorption heat can be determined by Equation (19):

(19) $$\Delta H_{sorp}=\Delta H_{H_{2O.ad}}^{f}-\Delta H_{H_{2O,gas}}^{f}$$

It is difficult to calculate the energy released from the electrochemical reactions within a polymer fuel cell. In general, the amount of energy and electricity can be calculated based on the variations in the enthalpy or entropy of the electrochemical systems. Here, the entropy variations were considered to calculate the heat released during the reaction.

The amount of entropy corresponding to species i at the temperature of T and pressure of P can be obtained by Equation (20):

(20) $$S_{i}\left(T,P\right)=S_{i}^{O}+\int_{T_{0}}^{T}\frac{C_{p,i}}{T}dT+\int_{P_{0}}^{P}(-\frac{\partial v_{i}}{T})dP$$

where $v_{i}$ is the specific volume corresponding to species *i* and $S_{i}$ denotes the absolute entropy corresponding to species *i* at $T_{0}=298.15$ K and $P_{0}=1$ bar (standard conditions).

Although the entropy changes associated with the overall reaction in a polymeric fuel cell are certain, it is difficult to determine which part of this entropy is related to the cathode or anode half-reactions. Thermodynamic equilibrium equations in anode and cathode are based on entropy values of charged species which were approximated in previous works. In other words, it is impossible to make a cationic solution without any anionic particles, or vice versa. Therefore, it is difficult to determine the entropy of ions. By assuming that the produced water is in liquid form, the absorption/desorption heat of water molecules at the surface of the electrodes should be taken into account. The total amount of heat generated or consumed in the cathode and anode flow channels can be determined using the following equations, respectively [36,37,38,39,40]:

(21) QtotalC=Qsorpdesorpc+Qcond/evapC+QreacC+Qactc+Qconvc

(22) Qtotala=Qsorpdesorpa+Qcond/evapa+Qreaca+Qconvc

#### 2.3.5. Other Equations

Upon passing through the flow channels, the fluid loses a part of its energy due to the fluid-channel wall friction. The fluid pressure drop within the anode and cathode flow channels can be determined by the so-called Darcy–Weisbach Equation [40]:

(23) $$\Delta P_{f}=C_{f}\frac{L}{dh}\frac{{pV}^{2}}{2}$$

where $C_{f}$ is the friction coefficient, L shows the flow channel length, V denotes the fluid velocity, d is the hydraulic diameter of the flow channel, and *P* represents the fluid pressure drop. The friction coefficient, $C_{f}$, is a function of the dimensionless Reynolds number and can be obtained through the following equation:

(24) $$C_{f}=\{\begin{array}{c} c/Re\\ 0.079Re^{-1/4} \end{array},\;Re\leq 2000$$

(25) $$Re=\frac{{\mathrm{pvd}}_{h}}{\mu },\;Re\geq 4000$$

where μ represents the fluid viscosity and c is a constant dependent on the dimensions of the flow channel. To obtain the friction coefficient in the transient regime, a linear relationship between the friction coefficient can be used at Re=2000 and Re=4000 [39].

The total values of the molar, mass, and volumetric flow rates of the gaseous phase in the cathode and anode flow channels can be obtained by the following equations, respectively:

(26) $${\dot{n}}_{T}=\sum_{i=1}^{N}{\dot{n}}_{1}$$

(27) $${\dot{m}}_{T}=\sum_{i=1}^{N}{\dot{n}}_{1}{MW}_{i}$$

(28) $${\dot{Q}}_{T}=\frac{{\dot{m}}_{T}}{p_{\mathrm{mix}}}$$

(29) $$V_{\mathrm{fluid}}=\frac{{\dot{Q}}_{T}}{A_{\mathrm{channel}}}$$

where ${\dot{m}}_{T}$, ${\dot{n}}_{T}$, and ${\dot{Q}}_{T}$ represent the total values of the molar rate, mass rate, and volumetric flow rate of the gas entering the flow channels. Furthermore, $n_{i}$ shows the molar rate of the gas phase, while ${MW}_{i}$ indicates the molecular weight of the gas phase. Additionally, $p_{\mathrm{mix}}$ shows the density of the gas mixture, $A_{\mathrm{channel}}$ is flow channel cross-section, $V_{\mathrm{fluid}}$ represents the velocity of gas phase within the flow channel, and *N* stands for the number of gas phase components. The relative humidity in the anode and cathode flow channels can be determined by Equations (30) and (31):

(30) $$RH=\frac{P_{H2O}}{p^{sat}}$$

where $P_{H2O}$ and $p^{sat}$ are the partial pressure and saturation pressure of water vapor, respectively. The hydraulic diameter for the gas flow channels can be obtained by Equation (31).

(31) $$d_{h}=\frac{2ab}{a+b}$$

Regarding the rectangular shape of the flow channel cross-section, *a* and *b* in Equation (31) show the dimensions of this rectangle [38,39,40].

### 2.4. Solution

In this research, both the anode and cathode of the system encompassed two phases of gas and liquid. Moreover, the flow regime in the flow channels was of the steady type. On the cathode side, the gas phase is a mixture of oxygen, nitrogen, and water vapor while the gas phase of the anode side included hydrogen and water vapor. The liquid phase was water on both anode and cathode sides. The cross-sectional dimensions of the gas flow channels were in the millimeter range. In this modeling, different types of heat generation-consumption models (e.g., half-reactions heat, activation heat, absorption/desorption heat) in the anode and cathode, as well as water flood conditions, were considered to assess the performance of the fuel cell. The flow channels were meshed by the square-shaped elements and the governing equations were numerically discretized in a steady-state using the finite difference method and ultimately solved using MATLAB software. The bipolar plates of the fuel cell had several gas flows channels, called grooves, to which the hydrogen and oxygen gases entered.

**Figure 4:**
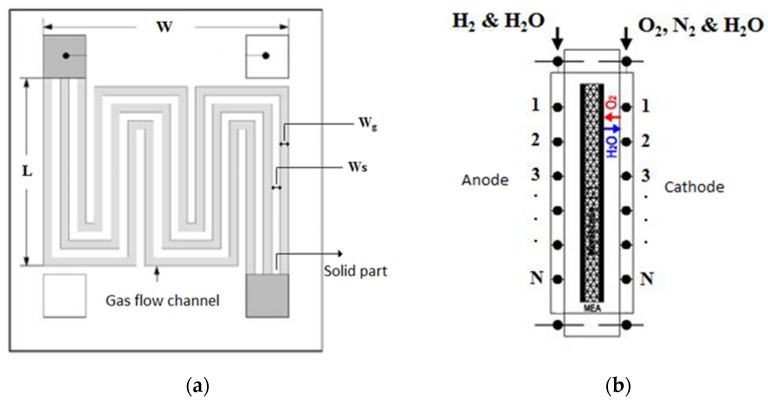
The bipolar plates consist of a number of gas flow channels in the fuel cell: (**a**) schematic of a bipolar plane with grooves; (**b**) A schematic of a flow channel node.

The flow channels were assumed to be spiral. Figure 4a shows a schematic representation of a bipolar plane with grooves. The width and height of the bipolar plate are indicated by *W* and *L*, respectively. The groove width and the width of the solid section are also shown by *Wg* and *Ws*, respectively. The cross-sectional area of the flow channels is rectangular. In this figure, this cross-sectional area is shown with the dimensions of *Wg* and *Hg*. For the numerical solution of the governing equations, the geometry of the problem should be meshed. Given that the cross-sectional dimensions of the flow channels are in the millimeter realm, the geometry of the problem can be simplified from a three-dimensional state into a one-dimensional one, due to the slight changes of different variables in the other two directions. By determining the number of nodes, the flow channels were meshed, and the governing equations were solved (Figure 4b). It should be noted that the number of nodes was proportional to the length of the flow channel, and the longer the flow channel length, the greater the number of these nodes. The minimum number of nodes is a number beyond which no significant change can be observed in the results.

As mentioned, in the modeling section, a mathematical model was proposed based on the governing mass conservation equations, thermal energy equations, species equations, and electrochemical equations to evaluate the transfer and electrochemical phenomena in a polymer fuel cell. In addition, some heat and cooling sources can be considered in a polymer fuel cell as follows:

- Water absorption/desorption at the GDL–membrane interfaces.
- Heat released/absorbed due to phase change of water in the GDL.
- Heat due to half-reaction entropy.
- Heat generated by cathode electrochemical activation.
- Convective heat transfer between gas flow within the channel and on its surface.

The following assumptions were considered in the modeling:

- The ideal gas mixture is considered in both anode and cathode gas flow channels.
- Homogeneous porosity and permeability of the GDP and their corresponding effective porosity and permeability parameters are defined.
- The thickness of the catalytic layer and GDL is considered very small in the micrometer range.
- Negligible voltage drop due to the catalytic layers and bipolar plates.
- The flow in the channels is assumed to be laminar. The simulation is performed one-dimensionally, due to the millimeter dimensions of the channels.
- The governing equations are assumed in a steady state.

### 2.5. Parameters Used in This Modeling

Table 1 reports a number of parameters used in this modeling and their types in terms of being constant or variable. Also, Table 2 shows the Values considered as Input Variables. Moreover, It should be noted that the list of symbols is shown in Abbreviations.

### 2.6. Laboratory Test and Polarization Curve

The flow scanning method can be used to study polarization curves. In the current sweeping method, the current between the upper and lower limits is swept at a certain speed and the potential is recorded. To activate the electrode-membrane assembly, single cells made at low currents are prepared and activated before the tests are performed. The connection between the membrane and the catalyst layer was well established. After the activation stage, polarization curves are obtained. After making the electrode–membrane assembly, in order to perform the fuel cell performance test, the prepared electrode–membrane assembly should be placed in a fuel cell unit to calculate the polarization of the fuel cell, including the prepared electrode–membrane assembly [31,39].

**Figure 5:**
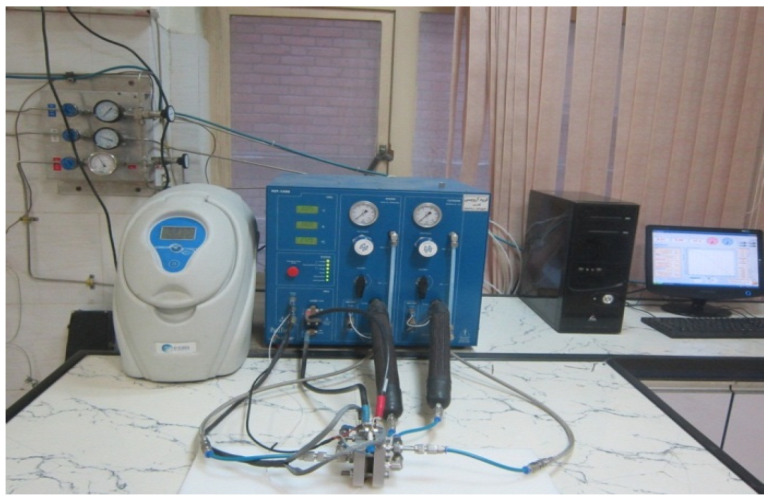
FCT-150s Fuel Cell Station.

In this project, a single fuel cell was built and tested. The polarization curve was obtained using a fuel cell test device (manufactured by the Biologic Company, Model FCT-150s) with a capacity of 250 watts (Figure 5). With the help of this device, various electrochemical tests, such as polarization and impedance, can be performed to evaluate the efficiency of the fuel cell.

## 3. Results and Discussion

### 3.1. Numerical Pattern Validation

**Figure 6:**
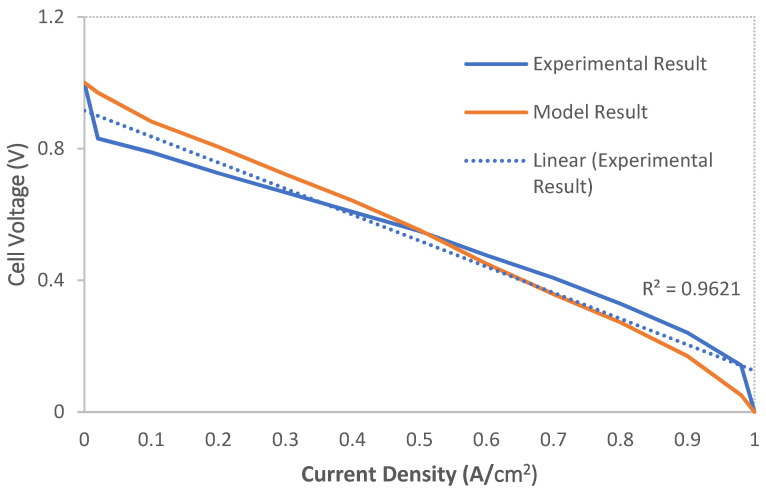
Cell voltage vs. current density (Model and Experimental results).

Figure 6 confirms the modeling results. In simulation research in the field of polymer fuel cells, validation by plotting cell voltage in terms of average current density is common [31,39]. As can be seen in the “Input Variables” section, the number of data/application input parameters is large. For this reason, in this study, the data/parameters of the program were included in the reported conditions for validation. Then, the program was executed, and the cell voltage diagram was drawn in terms of average current density. The general trend of this diagram was compared with the general trend of the cell voltage diagram in terms of average current density obtained from the laboratory results (Section 2.5). As can be seen from Figure 6, the general trend of these two graphs (modeling and laboratory results) is acceptably matched. At the initial and end current densities, the correlation between the model and laboratory results is less strong. Due to the activation-losses and ohmic-losses, the voltage drop is observed more rapidly [31,39].

### 3.2. Effect of Inlet Temperature

**Figure 7:**
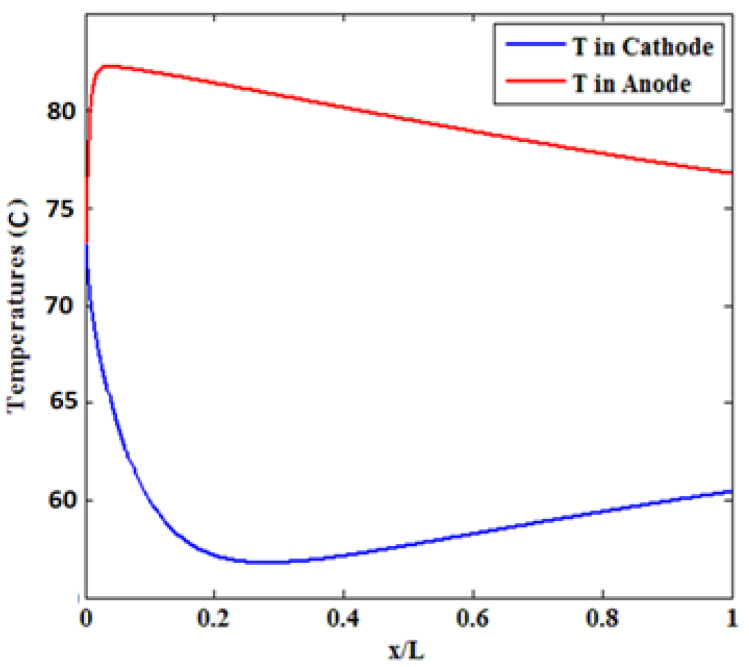
The temperature variations along the channel length.

The surface temperature of the bipolar plates and electrodes (both cathode and anode) can be considered constant. As the operating temperature of a polymeric fuel cell is between 65 and 120 °C, the temperatures of all parts of the fuel cell are considered to be in the same range [20]. Current density dramatically affects the cell temperature distribution, as the most important heat source in the polymer membrane cell is the heat of electrochemical reactions and the irreversibility of these reactions, which is proportional to the current density according to Equation (22). Since the local heat generation decreases along with the channel, the heat generation rate also decreases along the channel, and its maximum is close to the channel inlet. Therefore, the maximum temperature occurs near the inlet of the cathode channel. Figure 7 depicts the temperature variations along the channel length. By moving towards the end of the channel, the temperature decreases, and the maximum temperature in all sections occurs at the catalyst–membrane interface. The maximum temperature in the anode is equal to 83 °C, which decreases to 78 °C along the current channel. On the cathode side, the temperature shows a different behavior, such that it first decreases to 58 °C followed by a rise to about 62 °C. Therefore, as expected, the average temperature on the anode side is higher than that of the cathode. The resulting temperature variation is significant, especially when the cells are connected in series to form a bulky cell.

At moderate current densities, where ohmic drops are predominant in the fuel cell, temperature elevation raises the ionic conductivity of the membrane and improves cell performance. At high current densities, temperature increment increases the vapor pressure, hence, preventing the negative effect of flotation on cell performance. At very high temperatures (90 °C), although an increase in the cell temperature can reduce the transfer losses and increase the rate of electrochemical reactions, it can decrease the cell potential due to the slight increase in water vapor pressure and drying of the membrane. On the other hand, drying reduces ionic conductivity and increases thermal stresses, leading to a membrane rupture [24]. Therefore, the performance diagram was examined at 82 °C and cooler.

### 3.3. The Effect of Humidifying the Inlet Gases

The operating conditions of the polymer fuel cells require the humidification of inlet gas flow. In these cells, the relative humidity for the gases entering the anode and cathode flow channels is usually considered between 70% and 100%. The pressure of the inlet gases to the cathode and anode flow channels should be equal or have a slight difference as a high-pressure difference between the anode and cathode increases the possibility of damaging the fuel cell components on the cathode/anode side [21]. The fuel entering the cathode flow channel is a mixture of oxygen, nitrogen, and water vapor. In some fuel cell models, completely humidified air and fuel enter the anode and cathode channels to ensure hydration and minimize the ohmic loss of the membrane. In such cases, the gas inside the cathode channel can be supersaturated during cell operation, resulting in floating even at low current densities, in addition to imposing extra costs of inlet gas humidification equipment and power consumption [39].

**Figure 8:**
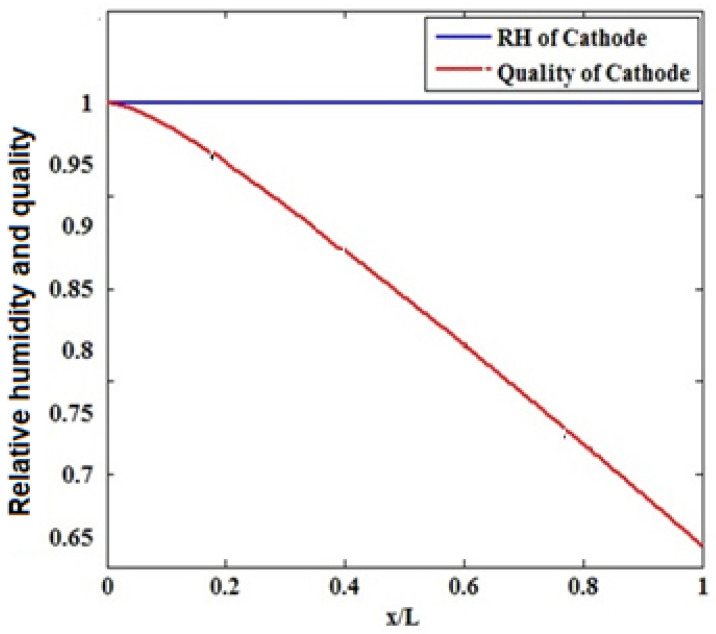
Relative humidity and quality of water vapor in the cathode flow channels.

The recent tendency of industries is toward the use of low relative humidity at the cathode inlet. Figure 8 shows the relative humidity and quality of water vapor in the cathode and anode flow channels. As observed, the relative humidity on the anode side sharply drops at its beginning along the flow channel followed by a milder decrease. However, the relative humidity on the cathode side is always saturated. Water is condensed into liquid when the water concentration exceeds the saturation level. For completely humidified inlet gases, condensation starts from the beginning of the channel, and liquid water is formed throughout the cathode gas diffusion layer. By blocking the pores of the gas diffusion layer, liquid water causes this layer to float. Floating prevents oxygen from reaching the reactant layer, decelerating the electrochemical reactions and dropping the cell performance [23,24,25,26,27,28,29,30,31,32,33,34].

**Figure 9:**
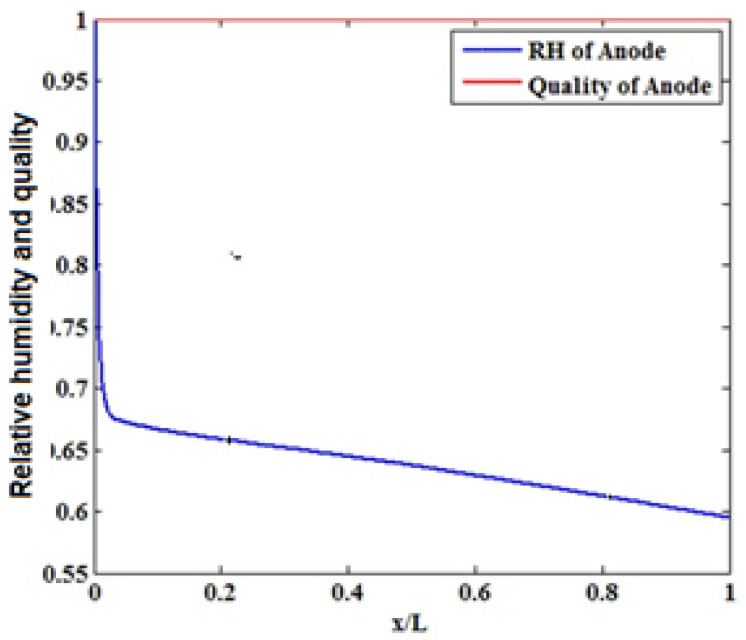
Relative humidity and quality of water vapor in the anode flow channels.

With an increase in current density, cell performance declined by increasing moisture content. By enhancing the inlet humidity, flotation occurs in a wider area of the gas diffusion layer of the cathode, although flotation occurs across the cathode gas diffusion layer when the inlet gases are completely wet Figure 9.

This phenomenon blocks the pores of the gas diffusion layer. Hence, less oxygen can reach the gas diffusion layer, resulting in a decrease in cell performance. As the cell power is the product of the voltage multiplied by the current density of the cell, the described phenomena have a similar influence on the power density diagram, and the maximum power can be achieved at poor cathode relative humidity [35,36,37,38,39].

### 3.4. Reynolds Number

**Figure 10:**
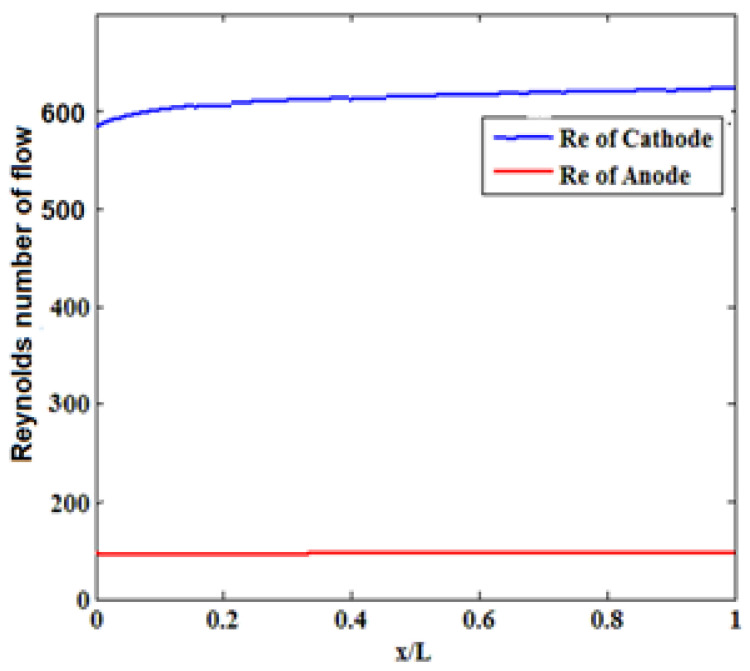
Reynolds number of flows.

The Reynolds number indicates the ratio of inertial forces to viscous forces due to fluid motion. Regarding the dependence of the flow turbulence or stratification on these forces, the Reynolds number can be used to determine the flow regime (stratified or turbulent). The size of fuel cells is predicted to decrease giving rise to a lower Reynolds number of flows in cooling channels. A reduction in the Reynolds number has a negative effect on the cooling of the cell. Based on Figure 10, the steady flow passes the anode side as no disturbance can be seen in the flow passing all channels on the cathode and anode sides. In all models, the difference between the maximum temperature and the minimum surface temperature decreases by increasing the inlet Reynolds. At high concentrations, ΔT tends to a specific value for all models, which depends on the Reynolds number.

## 4. Conclusions

The modeling proposed in this study considered different types of heat generation-consumption in the anode and cathode (such as half-reaction heat, activation heat, and absorption/desorption heat) and flooding conditions to evaluate the performance of the fuel cell. The flow channels were meshed by the square elements and the governing equations were numerically discretized under steady state using the finite difference method. Finally, the equations were solved using MATLAB software. The results of simulations indicate that:

The anode absorption/desorption heat, anode half-reaction heat, and cathode activation heat are positive, while negative values are obtained for the cathode absorption/desorption heat and cathode half-reaction heat.

The amount of anode absorption/desorption heat, anode half-reaction heat, and cathode activation heat decreased along the flow channel.

Evaluating the effect of relative humidity showed no significant changes in cathode relative humidity along the flow channel while the anode relative humidity decreases along the flow channel length.

The velocity in the membrane layer is significantly lower than that of the gas diffusion and reactant (cathode) layers, due to the smaller permeability coefficient of this layer. At the beginning of the channels, velocity is expanding.

## Figures and Tables

**Table 1:** Names and types of a number of parameters used in modeling.

Number	Variable Name	Parameter Type	Variable Symbols 1	Title 2	Title 3
1	Bipolar plate dimensions and groove dimensions	Constant	L_BP & W_BP	data	data
2	Number of nodes	Variable	N_s	data	data
3	The temperature of anode and cathode bipolar plate surface, membrane, and electrode surface	Variable	t_c_In & t_a_In		
4	Dry membrane weight, thickness, and porosity	Constant	M_m_dry & t_m & Phi		
5	The diffusion coefficient, anode, and cathode heat transfer coefficient	Constant	D_std_O_2__H_2_O & D_std_O_2__N_2_ & h_h_a & h_h_c		
6	Oxygen and nitrogen molar fraction in the inlet air, the total inlet pressure of the cathode and anode	Constant	Yo2_c_In & Yn2_c_In & P_c_In & P_a_In		
7	Average current density, relative humidity of cathode and anode inputs	Variable	I_ave & RH_c_In & RH_a_In		

**Table 2:** Values considered as ‘Input Variables’.

Title 1	Parameter	Amount
cell	∘*I*	100 (Am^−2^)
	β _2_	2.0
	*I_um_*	2.1 × 104 (Am^−2^)
cell	*E_cell_*	0.01–1.04 (v)
	*w*	1 (mm)
Flow channel	*h*	1 (mm)
	*w_s_*	1 (mm)
	*n_g_*	5
	*S_c_*	1.7
Flow channel	*S_a_*	1.1
	$T_{c}^{in}$	70 (°C)
Flow channel	$T_{a}^{in}$	70 (°C)
Flow channel	$RH_{a}^{in}$	1.0
Flow channel	$RH_{c}^{in}$	1.0
Flow channel	$P_{a}^{in}$	1.5 (atm)
Flow channel	$P_{c}^{in}$	1.5 (atm)
Gas flow distributor layer	$Y_{O2}^{in}$	0.21
Gas flow distributor layer	$t_{GDL}$	0.3 (mm)
Gas flow distributor layer	Φ	60%
Gas flow distributor layer	$K_{W}^{\circ }$	1 × 10^−10^ (m^2^)
Gas flow distributor layer	*dP_c_/dS*	−28.42 (Pa m^−1^)
Gas flow distributor layer	*k_c_*	1 (s^−1^)
Gas flow distributor layer	$s_{\delta }$	0
Membrane	*t_m_*	0.1275 (mm)
Membrane	*T_mem_*	70 (°C)
Membrane	ρ *_m,dry_*	2000 (kg m^−3^)
Membrane	*M_m,dry_*	1.1 (kg mole^−1^)
Bipolar plates	*W*	9.9 (cm)
Bipolar plates	*L*	9.9 (cm)
	*n*_s_	400
Other parameters	*I_ave_*	2000 (Am^−2^)
Other parameters	$T_{s}^{a}$	70 (°C)
Other parameters	$T_{s}^{c}$	70 (°C)
Other parameters	$T_{e}^{a}$	70 (°C)
Other parameters	$T_{e}^{c}$	70 (°C)
Other parameters	*D_o_*	5.5 × 10^−11^ (m^2^s^−1^)
Other parameters	$D_{O_{2}-H2O}^{std}$	0.36 × 10^−4^ (m^2^s^−1^)
Other parameters	$D_{O_{2}-n2}^{std}$	0.18 × 10^−4^ (m^2^s^−1^)
Other parameters	*h_a_*	25 (Wm^−2^k^−1^)
Other parameters	*h_c_*	25 (Wm^−2^k^−1^)

## Data Availability

The datasets used and/or analyzed during the current study are available from the corresponding author on reasonable request.

## Abbreviations

*L*	Bipolar plate height
*W*	Bipolar plane width
*W_g*	Groove width
*H_g*	Groove depth
*W_s*	The width of the solid part
*K_c*	Constant condensate/water evaporation rate
*I_ave*	Average current density
*S*	Stoichiometric coefficient
*T*	Temperature
*RH*	relative humidity
*P*	Pressure
*E*	Cell voltage
*GDL*	Gas penetration layer
*D*	Infiltration coefficient
*Ecell*	Cell voltage
*K*	Hydraulic permeability
$T_{c}^{in}$	Cathode inlet temperature
$T_{a}^{in}$	Anode inlet temperature
$P_{a}^{in}$	Anode inlet pressure
$P_{c}^{in}$	Cathode inlet pressure
*Ρ*	Density
*σ*	Surface tension
*ν*	Cinematic viscosity
$T_{s}^{c}$	Cathode surface temperature
$T_{e}^{a}$	Anode surface temperature
*T_mem_*	Membrane temperature
*t_m_*	Membrane thickness
$t_{GDL}$	The thickness of the gas penetration layer
$S_{a}$	anode stoichiometric coefficient
$S_{c}$	cathode stoichiometric coefficient
$A_{cell}$	cross-section
F	Faraday constant
$\dot{{\dot{N}}_{H_{2}}^{cell}}\;\mathrm{and}\;\dot{{\dot{N}}_{O_{2}}^{cell}}$	input molar flow rate to flow channels for hydrogen and oxygen, respectively
v	the vapor phase index
*R*	demonstrates the universal gas constant
$k_{w}$	permeability of liquid water in the GDL
$p_{w}$	the liquid water density
$M_{w}$	the water molecular weight
$E_{cell}$	output voltage of a cell
$\eta_{act},\;\eta_{ohm}$	the activation overpotential (v), ohmic overpotential (v)
$P_{H_{2}}$ and $P_{O_{2}}$	partial pressures related to hydrogen and oxygen (Pa)
$C_{O_{2}}^{cat}$	the oxygen concentration at the catalyst level
$C_{o_{2}}^{bulk}\left(x\right)$	the oxygen concentration in the flow channel
$D_{o_{2}-g}^{eff}$	the oxygen-effective diffusion coefficient in the gaseous mixture
$C_{p,mix}$	the gaseous mixture heat capacity
$k_{mix}$	the gas mixture thermal conductivity
$C_{f}$	the friction coefficient
μ	the fluid viscosity
${\dot{m}}_{T}$, ${\dot{n}}_{T}$, and ${\dot{Q}}_{T}$	the total value of the molar rate, mass rate, and volumetric flow rate of the gas entering the flow channels
$P_{H2O}$ and $p^{sat}$	partial pressure and saturation pressure of water vapor
